# The Factors Associated With Mutant Epidermal Growth Factor Receptor Positivity in Patients With Lung Cancer: A Study From a Tertiary Care Center in Pakistan

**DOI:** 10.7759/cureus.69631

**Published:** 2024-09-18

**Authors:** Talha Mahmud, Muhammad Idrees, Shazia Rafique, Rabbia Abbas, Iqra Anwar, Muhammad Saqib, Hala Talha, Muntaha Mehmood, Hadia Kashif

**Affiliations:** 1 Pulmonary Medicine, Shaikh Zayed Federal Postgraduate Medical Institute, Lahore, PAK; 2 National Centre of Excellence in Molecular Biology, University of the Punjab, Lahore, PAK; 3 Department of Pulmonology, Ittefaq Hospital, Lahore, PAK; 4 Department of Pulmonology, Shalamar Hospital, Lahore, PAK; 5 Economics, Forman Christian College University, Lahore, PAK

**Keywords:** adenocarcinoma, epidermal growth factor receptors, lung cancer, mutation, tyrosine kinase

## Abstract

Objective

In this study, we sought to elucidate the relationship between demographic and clinical factors and epidermal growth factor receptor tyrosine kinase (EGFR-TK) positivity in patients with advanced-stage lung cancer at a tertiary care center in Pakistan.

Methods

This analytical cross-sectional study was conducted from February 2020 to July 2023 at Shaikh Zayed Hospital, Lahore, Pakistan, in collaboration with the Centre of Excellence in Molecular Biology (CEMB), University of the Punjab. The study included 70 consecutive patients with advanced-stage lung cancer, and aimed to identify common EGFR mutations (Exon 19 deletion and Exon 21 L858R mutation), determine their frequency, and correlate EGFR-TK mutation positivity with clinical and non-clinical factors. Tissue acquisition and processing involved bronchoscopy, pleuroscopy, or percutaneous ultrasound-guided lung mass biopsy, followed by molecular analysis using polymerase chain reaction (PCR) extraction, amplification, and sequencing. The study employed convenient sampling and included adults aged over 18 years with histologically confirmed lung cancer.

Results

We enrolled 70 lung cancer patients, with a mean age of 60.87 years, and a male-to-female ratio of 1.4:1. The majority (61.4%) had adenocarcinoma, and 34.3% harbored a mutant EGFR-TK. EGFR mutations were more common in adenocarcinoma (91.7%) and associated with female non-smokers. Binary logistic regression analysis revealed that age <50 years and adenocarcinoma type were significant predictors of EGFR mutations. We found no significant associations with gender, smoking status, or other variables. These findings have implications for personalized treatment approaches in lung cancer patients.

Conclusions

Our study reveals a high frequency of occult EGFR mutations (Exon 19 deletion and Exon 21 L858R mutation) in non-small cell lung cancer (NSCLC) patients, particularly among male smokers and female non-smokers. These findings emphasize the importance of routine EGFR mutation testing to identify patients who may benefit from targeted therapies, ultimately improving treatment outcomes.

## Introduction

Lung cancer, or bronchogenic carcinoma, refers to malignancies that originate in the airways or lung parenchyma. Lung cancer is generally classified as either small cell (20% of cases, mostly central tumors with falling incidence) or non-small lung cancer (NSCLC), which accounts for the remaining 80% [1]. Besides proven causative agents like tobacco smoking, advancing age, chronic obstructive pulmonary disease (COPD), and exposure to chemicals like nickel, arsenic, chromium, asbestos, radon, etc., there is an individual genetic susceptibility to lung cancer and 10-15% of never-smokers who develop this disease may have a strong family history of lung cancer [2]. NSCLC treatment varies by stage, with surgery for stages IA-IIB, chemo-radiotherapy for stage III, and chemotherapy for stage IV [3]. Rapid advances in understanding the molecular pathogenesis of lung cancer have demonstrated the presence of different molecular abnormalities (genetic mutations) in some of these patients, leading to the categorization of lung cancer as part of a heterogeneous group of diseases.

Tumor growth and progression depend on cell surface membrane receptors like epidermal growth factor receptors (EGFR), controlled by tyrosine kinases (TKs) [4]. While the TK activity of EGFR is tightly controlled in normal cells, the genes encoding these receptors may have escaped from their usual intracellular inhibitory mechanisms in malignant cells [5]. Mutant EGFR is overexpressed in 10% of white and 40% of East Asian NSCLC patients, leading to uncontrolled tumor growth [6]. Binding to the EGFR triggers intracellular signaling events through at least three major pathways (the AKT, MAPK, and STAT pathways), leading to proliferation, invasion, angiogenesis, metastasis, and inhibition of apoptosis (uncontrolled tumor growth) [5,6]. Therapeutic targeting of these mutations has become the first-line standard therapy, with chemotherapy reserved for those who fail to respond to molecular treatments.

This study investigated demographic and clinical factors associated with mutant EGFR-TK positivity in patients with primary advanced-stage (IV) lung cancer. Tissue samples were obtained through bronchoscopy, pleuroscopy, or percutaneous lung biopsy and analyzed histologically and molecularly for EGFR-TK mutations, including Exon 19 deletion and Exon 21 L858R mutation. Our findings suggest a high frequency of occult EGFR mutations in NSCLC patients, particularly in male smokers and female non-smokers, highlighting the importance of routine evaluation for EGFR mutations to enable targeted therapeutic benefits.

## Materials and methods

Study design and setting

This analytical cross-sectional study was conducted between February 2020 and July 2023, with the approval of the Institutional Review Board (IRB No.1359), Shaikh Zayed Hospital, Federal Postgraduate Medical Institute, Lahore, Pakistan. It was carried out in the Department of Pulmonology at Shaikh Zayed Hospital in collaboration with the Department of Molecular Biology at the Centre of Excellence in Molecular Biology (CEMB), University of the Punjab, Lahore, Pakistan. A total of 70 consecutive patients with histologically confirmed advanced-stage (III-IV) lung cancer were included in the study. The objectives of this study were to identify the most common EGFR mutations, specifically EGFR Exon 19 deletion and EGFR Exon 21 L858R mutation, in patients with lung cancer, determine the overall frequency of mutant EGFR-TK mutations, and correlate EGFR-TK mutation positivity with various clinical and non-clinical factors. The study employed convenient sampling, and participants included adults over 18 years of age with histologically confirmed lung cancer.

Sample collection and processing

Tissue acquisition and processing were crucial steps in this study. Suspected lung cancer patients underwent various procedures, including bronchoscopy, pleuroscopy/medical thoracoscopy, or percutaneous ultrasound-guided lung mass biopsy, to obtain cancer tissue for analysis. Any biopsy specimens that revealed a diagnosis other than lung cancer through histopathology were discarded and replaced with a new sample to maintain a cohort of 70 patients. The obtained specimens were then categorized into two sets: one was formalin-fixed and sent for histopathological sub-typing, while the other was frozen in liquid nitrogen and transported to the CEMB laboratory for molecular analysis. This analysis focused on detecting the two most common EGFR mutations, Exon 19 deletion and Exon 21 L858R, using tissue polymerase chain reaction (PCR) extraction, amplification, and sequencing, strictly following recommended protocols to ensure accuracy and reliability.

Molecular analysis of the EGFR gene

The TK domain region of the EGFR gene was targeted for sequencing primers design, specifically exons 19 and 21, using Primer 3 software. Primers were designed from adjacent intronic regions to amplify these exons. DNA extraction from lung cancer biopsy samples was performed using the Thermo Fisher Scientific K1502 kit (Thermo Fisher Scientific, Waltham, MA). The extracted DNA was then amplified using PCR, optimized for reported mutations in exons 19 and 21, and the amplicons were verified on 1.5% agarose gel. Positive PCR products were purified and subjected to Sanger sequencing using automated APS bioscience equipment. Sequence reads were visualized using Chromas digital software and aligned using Geneiousprime software to create a consensus sequence, which was then mapped to the reference exon sequence retrieved from the ensembl.org database database. Multiple alignment tool in Clustal Omega was used to align sample sequences to their reference sequences. BLAST analysis was conducted to manually locate mutations, identified by nucleotide mismatches.

Statistical analysis

Data analysis was performed using SPSS Statistics version 20 (IBM Corp., Armonk, NY) to examine the relationships between various factors and the presence of EGFR-TK mutations. Numerical variables, such as age and smoking pack years, were summarized as mean ± standard deviation (SD), while categorical variables, including smoking status, past history, family history, occupational exposures, comorbid conditions, cancer type, and EGFR-TK presence, were described using frequency and percentage distributions. Binary logistic regression analysis was employed to investigate the impact of these factors on EGFR-TK mutations, including age, gender, smoking status, carcinoma type, past history of pulmonary TB, family history of cancer, occupational exposure, pulmonary fibrosis, cancer at other body sites, and active COPD. For all analyses, a p-value ≤0.05 was considered statistically significant with a confidence level of 95%.

## Results

Seventy lung cancer patients were enrolled in this study, comprising 59 (84.3%) inpatients and 11 (15.7%) outpatients at the Department of Pulmonology, Shaikh Zayed Hospital, Federal Postgraduate Medical Institute, Lahore Pakistan. The mean age of the patients was 60.87 ± 10.51 years [median: 61.5 years, interquartile range (IQR): 14 years (range: 35-83 years)]. The gender distribution was as follows: 29 (41.4%) females and 41 (58.6%) males.

Tumor tissue was obtained from patients suspected of having primary/metastatic lung cancer via various interventional procedures: including bronchoscopy (endobronchial biopsy) in 42 (60%) patients, ultrasound-guided trucut biopsy in 10 (14.28%) patients, and medical thoracoscopic pleural biopsy in 18 (25.71%) patients. Tumor samples were discarded if histopathology revealed a pathological diagnosis other than primary lung cancer, and replaced with a new patient to maintain a sample size of 70. The distribution of lung cancer subtypes among the 70 patients was as follows: adenocarcinoma (61.4%, n=43), adenosquamous carcinoma (11.4%, n=8), squamous cell carcinoma (8.6%, n=6), small cell carcinoma (14.3%, n=10), and large cell carcinoma (4.3%, n=3) (Table 1).

**Table 1:** EGFR-TK positivity and mutation with carcinoma type, gender, pulmonary comorbid conditions, and past history COPD: chronic obstructive pulmonary disease; EGFR-TK: epidermal growth factor receptor tyrosine kinase; TB: tuberculosis

Variable	Subcategory	Total participants (n=70), n (%)	Tested positive for EGFR-TK (n=24, 34.3%), n (%)	EGFR Exon 19 deletion (n=18), n (%)	EGFR Exon 21 L858R mutation (n=6), n (%)
Gender	Male	41 (58.6%)	14 (58.33%)	10 (71%)	4 (29%)
	Female	29 (41.4%)	10 (41.67%)	8 (80%)	2 (20%)
Type of carcinoma	Adenocarcinoma	43 (61.4%)	22 (92%)	16 (73%)	6 (27%)
	Adenosquamous carcinoma	8 (11.4%)	2 (6%)	2 (100%)	0 (0.0%)
Pulmonary comorbid condition	COPD	14 (20%)	4 (16.7%)	3 (75%)	1 (25%)
	Pulmonary fibrosis	7 (10%)	4 (16.7%)	4 (100%)	0 (0.0%)
	Cancer at any other site	3 (4.3%)	1 (4.2%)	1 (100%)	0 (0.0%)
Positive past history	Pulmonary TB	14 (20%)	3 (12.5%)	3 (100%)	0 (0.0%)
	Family history of cancer	4 (5.7%)	1 (4.2%)	1 (100%)	0 (0.0%)
	Occupational exposure	7 (10%)	2 (8.3%)	2 (100%)	0 (0.0%)

Among the 70 patients, 14 (20%) had a history of pulmonary tuberculosis in the past, and four (5.7%) had a family history of cancer in first-degree relatives. Comorbid conditions included COPD (20%, n=14), pulmonary fibrosis (10%, n=7), and cancer in another body site (4.3%, n=3). No patients had interstitial lung disease or a history of radiotherapy, except for one patient (1.4%) with a history of treated malignant disease (basal cell skin cancer). Seven patients (10%) had occupational exposure to potential carcinogens, including brake shoe manufacturing, construction, and plumbing.

In the cohort, 31 (44.3%) were non-smokers, while 39 (55.7%) had a history of smoking. The smoking subgroup consisted of 16 (22.9%) current smokers, 11 (15.7%) former smokers, six (8.6%) passive smokers, and six (8.6%) former passive smokers. Further inquiry revealed that 25 (64%) patients in the smoking subgroup were exclusive cigarette smokers, two (5%) were exclusive hookah (water pipe) smokers, and 12 (31%) had smoked both cigarettes and hookah. The mean smoking pack years was 34.28 ±14.55, with a median of 33 pack years. The correlation of smoking status among male and female subjects along with EGFR-TK-positive status is presented in Table 2.

**Table 2:** Smoking exposures among EGFR-TK-positive lung cancer subjects EGFR-TK: epidermal growth factor receptor tyrosine kinase

Smoking exposure	Subcategories	Total (n=70), n (%)	EGFR-TK-positive subjects, female (n=10), male n=14 (%), n (%)	
Smoker/non-smoker	Non-smokers	31 (44.3%)	7 (70%)	5 (36%)
	Smokers	39 (55.7%)	3 (30%)	9 (64%)
Current/former/passive/formerly passive smoker	Current smokers	16 (22.9%)	1 (10%)	4 (29%)
	Former smokers	11 (15.7%)	1 (10%)	3 (21%)
	Passive smokers	6 (8.6%)	1 (10%)	2 (14%)
	Formerly passive smokers	6 (8.6%)	0 (0.0%)	0 (0.0%)
Hookah/cigarette/combined smoker	Exclusive hookah smokers	2 (5.2%)	1 (33%)	0 (0.0%)
	Exclusive cigarette smokers	25 (64.1%)	2 (67%)	5 (56%)
	Combined cigarette and hookah smokers	12 (31.6%)	0 (0.0%)	4 (44%)

Among the 70 patients, 24 (34.3%) harbored a mutant EGFR-TK. Subtyping revealed 18 (75%) cases with EGFR Exon 19 deletion and 6 (25%) with EGFR Exon 21 L858R mutation. Notably, 22 (91.67%) of the EGFR-TK-positive patients had adenocarcinoma, and two (8.33%) had adenosquamous carcinoma. Table 1 presents the cross-tabulation of TK EGFR positivity and mutation with gender, carcinoma type, pulmonary comorbid conditions, and relevant history. Importantly, no cases with squamous cell carcinoma, small cell lung cancer, or large cell neuroendocrine carcinoma tested positive for EGFR mutations. Among the 24 patients who tested positive for EGFR-TK, the assessment of tumor expression revealed three (12.5%) with low expression, 13 (54.2%) with moderate expression, and eight (33.3%) with strong expression. Notably, most patients with strong expression levels (75%) were female non-smokers. The correlations between EGFR-TK positivity and mutation with various factors, including gender, carcinoma type, pulmonary comorbid conditions, and relevant history, are illustrated in Table 1.

Binary logistic regression analysis was performed to identify predictors of positive EGFR mutations. The model included age (<50, >50 years), gender, smoking status, carcinoma type, pulmonary TB history, family cancer history, occupational exposure, pulmonary fibrosis, cancer at other sites, and active COPD. The overall classification accuracy was 74.3%. The Omnibus Tests of Model Coefficients (p=0.002) and Hosmer-Lemeshow test (p=0.58) confirmed model fit. Nagelkerke R Square (0.451) indicated a significant proportion of variance explained by the predictors. The analysis revealed significant associations between EGFR mutation positivity and age group (<50 years: lower risk) and carcinoma type (adenocarcinoma: increased risk). No significant associations were found with gender, smoking status, or other variables (Table 3).

**Table 3:** Binary logistic regression analysis for EGFR mutation positivity *P<0.05 considered statistically significant; p-values calculated using binary logistic regression CI: confidence interval; COPD: chronic obstructive pulmonary disease; EGFR: epidermal growth factor receptor; TB: tuberculosis

Variable	Odds ratio	95% CI	P-Value
Gender			
Female vs. male	0.16	0.03-0.83	0.977
Age group			
>50 vs. <50 years	3.33	1.12-9.92	0.030*
Smoking status			
Non-smoker vs. smoker	2.23	0.42-11.60	0.340
Past history of TB			
No vs. yes	0.11	0.01-1.33	0.083
Family history of cancer			
No vs. yes	8.41	0.19-371.06	0.270
Occupational exposure			
No vs. yes	0.50	0.02-9.69	0.648
Pulmonary fibrosis			
No vs. yes	10.36	0.28-695.02	0.138
Active COPD			
No vs. yes	0.40	0.02-6.89	0.530
Type of carcinoma			
Other vs. adenocarcinoma	24.89	3.31-187.03	0.002*

Further analysis revealed that adenocarcinoma had a strong association with the expression of Exon 19 deletion mutation in EGFR-positive patients (p=0.04), but the gender, smoking status, occupational exposure, and family history of any cancer did not exhibit any significant association. On the other hand, Exon 21 L858R mutation expression in EGFR-positive cases showed no association with any of the above-mentioned study variables.

## Discussion

Lung cancer is among the most common cancers globally and is the leading cause of cancer mortality in men, and the second leading cause in women [7]. Our study population consisted of patients with advanced-stage lung cancers of both genders, with a majority of males (n=41, 58.6%), likely attributed to the high prevalence of smoking among males in our population [7,8]. Notably, only 11 (16%) out of 70 patients were outpatients who required daycare diagnostic bronchoscopy, whereas the majority (84%) were hospitalized in indoor wards due to advanced-stage lung cancer and associated complications such as post-obstructive pneumonia, lobar/pulmonary collapse, and/or malignant pleural disease/effusion. This reflects the high level of morbidity among this patient population. This finding is consistent with a comparative study that found patients with advanced-stage lung cancer required the most frequent and prolonged in-hospital treatment among different types of cancers [9].

Globally, cigarette smoking is the primary risk factor for lung cancer development, accounting for ~90% of all lung cancers [10]. The cumulative lung cancer risk among heavy smokers can be as high as 30%, compared to a lifetime risk of 1% or less in non-smokers [11]. In our study of 70 patients with primary lung cancers, 39 (55.7%) had exposure to tobacco smoke, either actively or passively. Among smokers, 16 (22.9%) were current smokers, and 11 (15.7%) were former smokers. Passive smoking has also been linked to an increased lung cancer risk. In our study, six (8.6%) patients were passive smokers, and six (8.6%) were formerly passive smokers. Notably, three patients with EGFR-positive lung cancer had lifelong exposure to secondhand smoke from their spouses. Hookah (water pipe) smoking has also been associated with an increased lung cancer risk [12]. Additionally, environmental and occupational exposures have been linked to pulmonary malignancies [13]. In our study, seven (10%) patients had occupational exposures that may have contributed to their lung cancer development, including brake shoe manufacturing, construction, and plumbing. Three of these patients were also active smokers, thus further increasing their risk [14].

Consistent with global trends, our study found that among all lung cancer patients, adenocarcinoma was the predominant histological type [15]. The rising incidence of adenocarcinoma is thought to be linked to the introduction of low-tar filter cigarettes in the 1960s. Additionally, 11.4% of patients had mixed histology adenosquamous carcinoma, an aggressive tumor with a poor prognosis [16]. Squamous cell lung cancer and large cell cancers were less common, accounting for 8.6% and 4.3% of cases, respectively, consistent with global patterns [10].

Guidelines recommend testing for driver mutations (e.g., EGFR, ALK, ROS1) in advanced NSCLC, particularly adenocarcinoma [17]. In our study, due to cost constraints and limited primer/kit availability, we focused on detecting the two most common mutations, Exon 19 deletion and Exon 21 L858R, in histopathologically confirmed tumors. This approach allowed us to provide genetic testing to patients at no additional cost. Moreover, the diagnosis of lung cancer in Pakistan is limited, with most patients in public hospitals receiving only a histopathological diagnosis. Advanced diagnostic techniques, such as immunohistochemistry, are largely confined to private laboratories, making them inaccessible to many patients. Molecular testing is also restricted, with only a few private laboratories offering tests for EGFR mutations, ALK rearrangements, and ROS1. Furthermore, next-generation sequencing for broad testing panels is not currently available in Pakistan due to cost constraints and limited accessibility [18].

Among all patients with positive EGFR-TK (n=24), an overwhelming majority (91.6%, n=22) had adenocarcinoma, while 8.3% (n=2) had adenosquamous carcinoma. In contrast, none of the patients with squamous cell lung cancers, large cell cancers, or small cell neuroendocrine tumors exhibited EGFR positivity, aligning with findings from international studies [19]. Guidelines from prominent medical organizations recommend analyzing adenocarcinoma tumors or metastases for EGFR and ALK mutations, particularly in patients with a history of light or never smoking [20]. Studies have shown varying frequencies of EGFR mutations in adenocarcinoma patients, with approximately 15% in the US and up to 51.4% in Asian populations [21]. Our study found a similar incidence of 51.16% EGFR mutations in adenocarcinoma patients, with an additional 25% of adenosquamous carcinoma patients also testing positive.

The PIONEER study reported a range of 22-62% EGFR mutations in adenocarcinoma patients across seven Asian regions [22]. Another Pakistani study found EGFR mutations in 29% of primary lung adenocarcinoma patients [23]. A retrospective study on advanced EGFR mutant lung carcinoma in Pakistani patients reported similar demographics to our study, with a majority of male patients (54.5%) and advanced stage IV NSCLC (90.9%) [15]. Notably, our study included a 35-year-old never-smoker with EGFR-positive adenocarcinoma, suggesting a possible genetic basis for the disease. We found that 75% of EGFR-positive patients had Exon 19 deletion and 25% had Exon 21 L858R mutation, differing slightly from another study's findings [15].

Lung cancer in non-smokers is a significant health concern, with distinct characteristics from smoking-related lung cancer [24]. Notably, adenocarcinomas are the most common type in never-smokers, particularly in Asian women [25]. Our study revealed a significant association between age over 50 years and EGFR mutations in primary lung cancers. Furthermore, we found that never-smoker males with adenocarcinoma/adenosquamous carcinoma have a 36% chance of EGFR positivity, with the majority harboring Exon 19 deletion. Similarly, female non-smokers with adenocarcinoma and EGFR-TK positivity accounted for 70%, with the majority exhibiting Exon 19 deletion. Interestingly, strong EGFR expression was more common in female non-smokers [26]. These findings align with previous studies, highlighting the importance of EGFR mutation testing in lung cancer patients, especially in never-smokers with adenocarcinoma [23].

Our study's findings further revealed significant associations between age group and EGFR-positive lung cancer, with individuals aged <50 years having a lower risk compared to those >50 years. Adenocarcinoma showed a strong association, increasing the risk of EGFR positivity compared to other carcinoma types. This aligns with international data suggesting lung cancer occurs in older individuals, likely due to smoking and latency of malignancy onset [27]. Adenocarcinoma was strongly associated with Exon 19 deletion mutation in EGFR-positive patients, consistent with an Indian study finding EGFR mutations in 32% of NSCLC cases, mostly Exon 19 deletions [28]. However, gender, smoking status, occupational exposure, and family cancer history showed no significant associations.

Among the 24 patients with EGFR-TK positivity, 12.5% had a history of pulmonary TB, 16.7% had active COPD, 16.7% had pulmonary fibrosis, and 4.2% had cancer in another body site. Notably, all patients with a history of pulmonary TB, pulmonary fibrosis, and cancer in another body site had adenocarcinoma with EGFR Exon 19 deletion. Three out of four patients with COPD had EGFR Exon 19 deletion, while one had EGFR Exon 21 L858R mutation. These findings align with previous studies, including a Taiwanese study that found a positive history of treated TB in 2.79% of lung cancer patients [29]. Additionally, studies have found that pulmonary fibrosis and active COPD are associated with an increased risk of lung cancer, with pulmonary fibrosis conferring an 8-14-fold increased risk and COPD increasing the risk by two to five-fold [30].

Limitations

Other recommended molecular-based tests, such as ALK, ROS1, KRAS, BRAF, and PD-L1, should also be part of the diagnostic workup in patients with NSCLC. However, due to resource constraints, our study only considered testing for the two most common EGFR mutations (Exon 19 deletion and Exon 21 L858R) in our cohort of patients with primary lung cancers, which was a limitation of our study.

## Conclusions

The frequency of common EGFR mutations, specifically Exon 19 deletion and Exon 21 L858R, is high in patients with NSCLC, predominantly adenocarcinoma, particularly in individuals younger than 50 years, male smokers, and female non-smokers. Therefore, EGFR mutation analysis should be included in the diagnostic workup to potentially gain prognostic benefits by therapeutically targeting these mutations with targeted drugs.
